# How does parental rearing patterns of children in upper primary school impact social withdrawal? A mediating effect of emotional regulation

**DOI:** 10.3389/fpsyg.2024.1382104

**Published:** 2024-07-05

**Authors:** Tao Yu, Zhengyu Ma, Yu Zhang

**Affiliations:** ^1^School of Educational Science, Shenyang Normal University, Shenyang, China; ^2^Shenyang Seventh Middle School, Shenyang, China; ^3^School of Art and Information Engineering, Dalian University of Technology, Dalian, China

**Keywords:** children in upper primary school, parental rearing patterns, emotional regulation, social withdrawal, child behavioral issues scale

## Abstract

**Introduction:**

The present study endeavors to elucidate the impact of emotional regulation and parental rearing patterns on the social development of children in the upper grades of primary school. A burgeoning body of literature suggests that these factors significantly influence children’s social adaptation and emotional well-being, yet a comprehensive examination of these relationships is warranted.

**Methods:**

Employing a cross-sectional design, this investigation utilized the Egna Minnen Beträffande Uppfostran (EMBU), Emotional Regulation Questionnaire for Children (ERQC), and Child Behavioural Issues Scale (CBCL) to assess a sample of 276 pupils across grades 4–6. The selection of these instruments allowed for a multifaceted evaluation of the constructs of interest.

**Results:**

A pronounced disparity in parental rearing practices, emotional regulation capabilities, and levels of social withdrawal was observed among the different grades, with grade 5 exhibiting the most pronounced effects. Parental emotional warmth demonstrated a significant positive correlation with children’s emotional regulation abilities, while punitive, rejecting, and preferential behaviors were inversely correlated. The study established that parental rearing practices indirectly influence social withdrawal through the mediating role of children’s emotional regulation, underscoring the complexity of this relationship.

**Conclusion:**

The results underscore the salient role of parental rearing and emotional regulation in the social development of children. The study contributes to the existing literature by providing a nuanced understanding of the mechanisms through which parenting styles and emotional competencies interplay to affect social withdrawal. Implications for educational practices and future research directions are discussed.

## 1 Introduction

Social withdrawal in children, characterized by a reluctance to engage in peer interactions and a preference for solitude, is a significant phenomenon that impedes effective social problem-solving and is correlated with diminished self-esteem. Such behaviors can foreshadow the development of anxiety, depression, and a spectrum of psychosocial challenges (Rubin et al., 2009). The importance of addressing these issues is underscored by the “Mental Health Action Plan for Children and Adolescents,” a collaborative initiative launched in 2019 by the Chinese National Health Commission and other governmental bodies, aimed at fostering mental health development and implementing preventive interventions for cognitive and behavioral issues among young people.

Upper primary school children, navigating the onset of puberty, often seek autonomy and may experience increased social withdrawal and mental health vulnerabilities as they strive to detach from parental oversight (Wood et al., 2017). This developmental stage, critical for the emergence of self-identity and social competence, underscores the necessity of investigating the determinants of social withdrawal during these formative years (Wood et al., 2017).

Ecosystem theory posits that family dynamics exert a profound and enduring influence on individual growth and development (Chang, 2003). Parental rearing styles, ranging from emotional warmth to punitive and rejecting behaviors, have varying impacts on children’s social development (Kato et al., 2020a). An environment marked by parental understanding and emotional support is conducive to lower incidences of social withdrawal, whereas over-surveillance and rejection can exacerbate social disengagement and communication deficits (Ike et al., 2020; Li et al., 2020). Self-Determination Theory (SDT) by Deci and Ryan (2000) adds another layer by positing that fulfilling three basic psychological needs - autonomy, competence, and relatedness - is crucial for children’s well-being and social development. Authoritative parenting fosters these needs, while less supportive styles can hinder them, potentially contributing to social withdrawal.

The burgeoning body of research underscores the pivotal role of emotional regulation in nurturing positive affective experiences, bolstering mental health, and enhancing interpersonal interactions (Morris et al., 2017). Emotional regulation—the capacity to modulate one’s emotional responses—is a critical adaptive skill that facilitates the management of adverse behaviors and cognitions in diverse situations (Bonanno and Burton, 2013). Children endowed with robust emotional regulatory skills exhibit greater self-regulation and socially adaptive conduct. Parental rearing practices significantly influence the development of these emotional competencies, with parental behaviors shaping children’s social development through their emotional regulatory abilities (Eisenberg et al., 2003). Cultivating emotional regulation in children not only assists in navigating negative emotions but also fosters the establishment of healthy interpersonal relationships and mitigates the risk of social withdrawal (Eisenberg et al., 2010).

Therefore, this study builds upon this foundational research to elucidate the interplay between parental rearing, emotional regulation, and social withdrawal. By examining these constructs, we aim to expand the scholarly discourse and offer insights that may inform strategies for reducing social withdrawal among children.

The subsequent sections of this paper are organized as follows: Section 2 synthesizes existing literature to establish a conceptual framework. Section 3 delineates the methodology, including the estimation techniques, sample selection, and data sources. Section 4 presents the empirical findings and an analysis of their implications. Section 5 concludes with a summary of the study’s contributions and suggestions for future inquiry.

## 2 Literature review

### 2.1 Parental rearing patterns

Parental rearing patterns are integral to the emotional regulation of children, a concept that has been subject to extensive scholarly inquiry. The family environment is the initial and most influential context for a child’s development, where many psychological attributes are shaped by the family dynamics (Zhang and Wang, 2020). Gottman’s seminal work (Gottman et al., 1996) has laid the groundwork in the domain of emotional parenting, emphasizing the pivotal role of parental responsiveness to children’s emotional states and the consequential impact on emotional regulation. His insights suggest that parents who provide timely and affirmative responses cultivate specific corrective behaviors and emotional cognitions that significantly shape a child’s regulatory abilities. Basso et al. (2019) extend this discourse by examining the ripple effects of parental rearing on the emergence of psychopathological indicators, particularly the interplay between rearing behaviors and the evolution of early maladaptive schemas (EMS). Their research underscores how certain rearing practices can either mitigate or exacerbate the development of these schemas, which in turn influence personality and behavioral trajectories. Adonteng-Kissi (2020) contributes to this dialog by exploring the alignment—or potential conflicts—between children’s rights and traditional child-rearing norms in Ghana, particularly within urban and rural settings. Maury et al. (2020) offer an evolutionary perspective, hypothesizing that the temporal allocation of parental care is intricately linked to reproductive success, suggesting an optimized approach to offspring rearing. Berbel-Filho et al. (2020) delve into the impact of environmental enrichment on the behavioral and epigenetic profiles of both parents and offspring in a species of fish, providing a novel lens through which to view the long-term effects of rearing environments. Wu et al. (2021) explore the nexus between parental rearing patterns and the development of interpersonal competencies among deaf university students in China, mediated by Theory of Mind (ToM), highlighting the cognitive mechanisms that underpin social adaptation.

### 2.2 Emotional regulation

Bronfenbrenner’s ecological systems theory brings into sharp focus the interplay between individuals and their environments, yet the intrinsic physiological underpinnings of children’s emotional regulatory capacities cannot be overlooked. Empirical evidence has consistently demonstrated a close relationship between personality traits and emotional regulation strategies (Matsumoto, 2006; Yuan et al., 2012). Children with less emotional responsiveness are often challenged in modulating their emotions, predisposing them to heightened anxiety. As cognitive research advances, cognition is recognized as the bedrock of emotional regulation, particularly with respect to the development of executive functions in children. Diamond (2013) illustrates that the emergence of working memory at 6 months marks a critical developmental milestone, enabling children to maintain and manipulate simple information. This underscores the interplay between genetic predispositions and cognitive growth in shaping emotional regulatory mechanisms.

There is evidence to suggest that the somatosensory cortex could be a target for treating certain mental disorders. Kropf et al. (2019) contribute to our understanding of the neural correlates of emotional regulation by elucidating the anatomical and functional roles of the somatosensory cortex. Morie et al. (2019) propose a mediating role for emotional regulation and alexithymia in the association between autistic traits and the severity of anxiety and depressive symptoms. Several studies have shown a link between emotional regulation and overweight or obesity in individuals with eating disorders. Casagrande et al. (2020) investigate the potential linkages between emotional dysregulation, alexithymia, and the comorbidity of overweight and obesity within the context of eating disorders. Thomas and Zolkoski (2020) employ path analysis to dissect the complex relationship between emotional intelligence, emotional regulation, resilience, and perceived stress among undergraduates, revealing resilience as a significant predictor of stress mitigation.

### 2.3 Social withdrawal

In the discourse on social withdrawal, Repetti (1989) scrutinizes the everyday variability inherent in marital behaviors, particularly social withdrawal and the expression of anger, as reflective of the day-to-day venture load encountered in occupational settings. Within this context, supportive spouses may mitigate the adverse outcomes of minor stressors by facilitating their partner’s social withdrawal. Rubin et al. (1993) delve into the realms of childhood social withdrawal, inhibition, and shyness, exploring their underpinnings and implications for early development. Boivin et al. (1995) assess the predictive power of social withdrawal, alongside peer rejection and victimization, in forecasting loneliness and depressive affect over time. They posit that social withdrawal’s contribution to subsequent loneliness is mediated by negative peer experiences (Eisenberg et al., 2000). Rubin et al. (1995) further examine the role of childhood social withdrawal and aggression as harbingers of adolescent maladaptation (Chang et al., 2003), providing a comparative analysis of the social and emotional outcomes associated with these behaviors (Bowker et al., 2016). Moreover, Rubin and Coplan (2004) offer an expansive perspective on the causes, correlates, and consequences associated with solitude, highlighting the complex interplay between temperamental shyness and the developmental trajectory of social withdrawal. Booth-Laforce and Oxford (2008) identify the preschool period as a critical juncture where shy temperament and inhibitory control strategies significantly influence the emergence and mitigation of withdrawal behaviors.

The lack of social interplay in childhood might also end result from a variety of causes, together with social worry and anxiousness or a desire for solitude. The study by Rubin et al. (2009) aims to clarify the conceptual and methodological landscape surrounding social withdrawal, identifying predictors, correlates, and consequences specific to toddler and early adolescent social withdrawal, and presenting a developmental framework that delineates the pathways to and from social withdrawal in childhood. Pérez-Edgar et al. (2010) extend this inquiry through a longitudinal examination of the familial and attentional biases that contribute to social withdrawal in adolescence, revealing that attention bias to danger moderates the relationship between childhood behavioral inhibition and adolescent social withdrawal.

In recent studies, Desjardins et al. (2020) investigate the longitudinal associations between social withdrawal and symptoms of anxiety/depression in post-brain tumor surgery, considering the interplay of medical, demographic, and personal factors. Tan et al. (2020) aim to ascertain the mediating role of loneliness in the relationship between social anhedonia and diminished social functioning. Meanwhile, the phenomenon of pathological social withdrawal, termed ‘hikikomori’ in Japan, has garnered increased attention as a complex and challenging behavior. Kato et al. (2020b) propose a conceptual model to explore the potential reciprocal relationships between pathological social withdrawal and internet dependency, suggesting a dual-directional influence. Aman et al. (2020) provide a comprehensive analysis of the Aberrant Behavior Checklist’s aspect structure in individuals with Fragile X syndrome (FXS), employing both exploratory and confirmatory methods to elucidate the distinct dimensions of social withdrawal present in this population. Rubin and Chronis-Tuscano (2021) evaluate the unique theoretical and conceptual bases that led to the first lookup software devoted to the developmental find out about of social withdrawal (the Waterloo Longitudinal Project). They additionally describe correlates (e.g., social and social-cognitive incompetence), precursors (e.g., dispositional characteristics, parenting, insecure attachment), and penalties (e.g., peer rejection and victimization, poor self-regard, anxiety) of social withdrawal, and talk about how the find out about of this kind of withdrawal led to a novel intervention (Ferrara et al., 2020; Scheer and Poteat, 2021; Masi et al., 2023).

Despite the wealth of research, the current study identifies a gap in understanding the impact of emotional regulation and parental rearing patterns on social retardation in upper primary school children. To address this, we employed the Egna Minnen Beträffande Uppfostran (EMBU^1^), Emotional Regulation Questionnaire for Children (ERQC), and Child Behavioural Issues Scale (CBCL) to survey 276 pupils in grades 4–6. Findings reveal significant differences in parental rearing, emotional regulation, and social retardation among the surveyed children, with fifth graders exhibiting lower levels of emotional warmth and higher instances of social withdrawal. Furthermore, the study indicates a positive correlation between parental emotional warmth and children’s emotional regulation abilities, while parental punishment, rejection, and preferential treatment are negatively associated with these abilities. The influence of parental factors on social withdrawal is suggested to be indirectly mediated through the emotional regulation capabilities of the children.

## 3 Methodology

### 3.1 Questionnaire and scale design

#### 3.1.1 EMBU

We use the questionnaire Jiang et al. (2010) revised in 2010, which consisted of 66 projects, each with four-level rating criteria, divided into two sub-questionnaires, the father-reeducation questionnaire and the mother-reeducation questionnaire. The father-rearing method Questionnaire is divided into six factors, (1) emotional warmth and understanding, (2) punishment and severity, (3) excessive interference, (4) preference for being tried, (5) rejection and denial, and (6) overprotection (Table 1). The mother-rearing questionnaire has five factors: emotional warmth and understanding, excessive interference and protection, rejection and denial, punishment and severity, and preference for being tested. In this study, due to the significant difference between the number of only and non-only children, this dimension will be preferred to be tested.

**Table 1:** Variables characters and items.

Parents	Characters	Measurement items
Father	Emotional warmth and understanding	2, 4, 6, 7, 9, 15, 20, 25, 29, 30, 31, 32, 33, 37, 42, 54, 60, 61, 66
	Punishing and severe	5, 13, 17, 18, 43, 49, 51, 52, 53, 55, 58, 62
	Excessive interference	1, 10, 11, 14, 27, 36, 48, 50, 56, 57
	Preference	3, 8, 22, 64, 65
	Reject and denial	21, 23, 28, 34, 35, 45
	Excessive protection	12, 16, 39, 40, 59
Mother	Emotional warmth and understanding	2, 4, 6, 7, 9, 15, 25, 29, 30, 31, 32, 33, 37, 42, 44, 54, 60, 61, 63
	Excessive interference and protection	1, 11, 12, 14, 16, 19, 24, 27, 35, 36, 41, 48, 50, 56, 57, 59
	Reject and denial	23, 26, 28, 34, 38, 39, 45, 47
	Punishing and severe	13, 17, 43, 51, 52, 53, 55, 58, 62
	Preference	3, 8, 32, 64, 65

#### 3.1.2 Emotion regulation questionnaire for children

This study uses a revised Chinese version of the Emotion Regulation Questionnaire for Children (ERQC), which Shields and Cicchetti prepare. The questionnaire consists of 24 items and is divided into two dimensions: negative and emotional regulation, two dimensions of internal consistency confidence of 0.96 and 0.83, respectively, and the overall internal consistency confidence of 0.69.

#### 3.1.3 Child behavior check list

We use the social retardation dimension of the Chinese standardized version of the Child Behavior Check List (CBCL), which Achenbach compiled. The CBCL, which uses the Child Behavior Problem Scale developed by Achenbach, is the social retardation dimension of China’s standardized version. The scale tests the social abilities and behaviors of children aged 4–16. The test is based on parents’ assessment of the child’s situation within 6 months. The higher the score, the greater the social retreat of children.

Parents are assessed according to the child’s situation within 6 months, using three points to calculate the total score finally. The Cronbach’s α coefficient in the measurement is 0.823, and the retest reliability of previous studies is 0.876 and has good validity.

### 3.2 Sampling and procedure

In this study, we investigate students in grades 4–6 who study in a primary school in Liaoning Province, in which the age range was 10–12 years. We test the student’s engagement in learning with no personal privacy issues involved. Three classes were randomly taken from each grade to examine their home-rearing methods, emotional regulation, and social retreat levels. From mid-March to mid-April 2023, a total of 300 paper questionnaires were issued, and 278 were recovered, with an effective recovery rate of 92.66%.

In order to ensure scientific, systematic, reliable, and representative data, we eliminate the questionnaire that belongs to these situations,

1. All questionnaire items select the same option or select less than five different options.
2. Questionnaire items are not completed, or some options are not filled in.
3. Questionnaire data of respondents who answered questions less than 1 min.

Finally, we obtained a final valid sample of 278 for subsequent data analysis.

### 3.3 Statistical methods and tools

We employed SPSS 25.0 for preliminary data analysis, utilizing descriptive statistics to summarize the central tendencies and dispersion of our variables. To ensure the assumptions of parametric testing were met, we examined the normality of the distribution of our measurement items through skewness and kurtosis assessments. For inferential statistics, we conducted the following analyses: Independent samples *t*-tests to discern mean differences in emotional regulation and social withdrawal between distinct demographic groups. One-way ANOVA to investigate the variance in social withdrawal and emotional regulation across different grades, treating grade level as the independent variable. Factorial ANOVA to explore the interaction effects between parental rearing patterns and emotional regulation on social withdrawal. Given the hypothesized mediating role of emotional regulation, we utilized AMOS 23 for structural equation modeling (SEM). SEM allowed us to rigorously test the proposed mediation model, estimating both direct and indirect effects within the framework of our theoretical hypotheses. We selected these analyses to align with the study’s objectives, providing a comprehensive examination of the relationships between parental rearing, emotional regulation, and social withdrawal.

## 4 Data analysis

### 4.1 Description statistics

We measure variable items with descriptive statistics, including sex and frequency, and account for the number. Table 2 illustrates the background information of the children’s families in this study.

**Table 2:** Description statistics.

Items	Categories	Frequency	Percentage (%)
Sex	Male	136	48.92%
	Female	142	51.08%
Grade	4th grade	83	29.86%
	5th grade	103	37.05%
	6th grade	92	33.09%
Single-parent family	Yes	117	42.11%
	No	161	57.89%
Parental education level	Student of bachelor’s degree courses (programs) or student of baccalaureate courses (programs) and above	25	8.99%
	Student of associate degree courses (programs) or junior college student	88	31.65%
	Junior high school and below	165	59.36%
Overall		278	100.00%

### 4.2 Differences in parenting style, emotional modulation, and social retreat among children in the upper grades of primary school

#### 4.2.1 Grade differences in the mode of parental rearing patterns of children in the upper grades of primary school

In order to analyze the differences between the fourth and sixth grades in the form of parental rearing patterns, the results of this study are shown in Table 3.

**Table 3:** Grade differences in the parental rearing patterns in the upper grades of primary school.

Items	4th grade (*n* = 83)	5th grade (*n* = 103)	6th grade (*n* = 92)	*F*
Excessive interference (F)	19.13 ± 4.95	21.28 ± 4.77	20.75 + 4.86	4.74^**^
Rejection (F)	10.24 ± 3.70	11.22 ± 4.06	9.84 ± 3.64	3.42^*^
Preference (F)	10.98 ± 3.03	12.18 ± 2.64	11.20 ± 3.07	4.68^**^

*p < 0.05, **p < 0.01, ***p < 0.001.

The results show that there are significant grade differences in the three dimensions of family upbringing, denial, and overprotection. After examination (LSD), it was found that the fifth grade was significantly higher than the fourth and sixth grades, and the fourth grade was significantly higher than the sixth grade. In the rejection dimension, grade five is significantly higher than grade six; In the overprotection dimension, grade five is significantly higher than grades four and six.

#### 4.2.2 Grade difference test of emotional regulation and social withdrawal in upper primary school children

In order to analyze the grade differences in emotional regulation and social withdrawal in children in 4th, 5th, and 6th grades, we perform a univariate ANOVA analysis of children’s emotional regulation ability and social withdrawal, and the results are shown in Table 4.

**Table 4:** Grade differences in social withdrawal of children in upper primary grades.

Items	4th grade (*n* = 83)	5th grade (*n* = 103)	6th grade (*n* = 92)	*F*
Emotional regulation	41.07 ± 8.03	36.43 ± 7.73	40.91 ± 7.06	11.66^**^
Social withdrawal	8.14 ± 6.45	13.68 ± 6.89	10.18 ± 6.33	17.01^**^

*p < 0.05, **p < 0.01, ***p < 0.001.

As can be seen from Table 2, there are significant grade differences in children’s emotional modulation ability and social withdrawal. After LSD (Least-Significant Difference), we found that the fourth and sixth grades were significantly higher than the fifth grade, the fifth grade was significantly higher than the sixth grade, and the sixth grade was significantly higher than the fourth grade.

### 4.3 Parental rearing patterns, emotional regulation and social withdrawal of children in upper primary schools

The correlation among the three variables of parental rearing patterns, emotional regulation and social withdrawal of children in the upper grades of primary school was tested by Pearson accumulation correlation analysis. The results are shown in Table 5.

**Table 5:** Analysis of parental rearing patterns, emotional regulation and social withdrawal.

Items	Emotional warmth	Punishment	Excessive interference	Preference	Rejection	Emotional regulation	Social withdrawal
Emotional warmth	1						
Punishment	−0.267^**^	1					
Excessive interference	0.268^**^	0.470^**^	1				
Preference	0.028	0.095	0.083	1			
Rejection	−0.284^**^	0.790^*^	0.471^**^	0.122^*^	1		
Emotional regulation	0.125^**^	−0.143^*^	−0.086	−0.219^*^	−0.224^**^	1	
Social withdrawal	−0.047	0.129^*^	0.117	0.121^*^	0.168^**^	−0.444^**^	1

*p < 0.05, **p < 0.01, ***p < 0.001.

As can be seen from Table 3, there is a significant correlation between the various dimensions in the three scales of parental rearing patterns, emotional regulation, and social withdrawal of children in the upper grades of primary school. The emotional warmth of parents is positively correlated with the emotional regulation ability of children. Parental punishment, preference, and rejection are significantly negative correlations with emotional regulation. There is a significant positive correlation with social retreat and a significant negative correlation between emotion regulation and social retreat.

### 4.4 Mediation effect test

#### 4.4.1 The mediating role of emotional regulation between parental punishment and social withdrawal

We use Model 4 in the SPSS macro-program to test the mediation between parental punishment and social withdrawal, as shown in Table 6.

**Table 6:** Test results of the mediation model of emotion regulation between parental punishment and social withdrawal.

Predictors	Outcome variables	Fit		Sig.	
		*R* ^2^	*F*	*t*	β
Parental punishment	Social withdrawal	0.02	4.66	2.16*	0.07
Parental punishment	Emotional regulation	0.02	5.80	−2.41**	−0.09
Emotional regulation	Social withdrawal	0.20	34.69	−7.98**	−0.38
Parental punishment		0.02	4.66	1.22	0.04

*p < 0.05, **p < 0.01, ***p < 0.001.

The results show that parental punishment is positive to predict social retreat ( β=0.07 , p<0.05 ) and negative to predict emotional regulation ( β=0.09 , p<0.01 ). Parental punishment and emotion regulation ability simultaneously enter the equation, emotion regulation ability ( β=−0.38 , p<0.01 ) negative prediction of social retreat.

The bias-corrected percentile Bootstrap test shows that, As shown in Table 5, the confidence interval of 95% of the social retreat path coefficient for parental punishment is 0.02–0.09, including 0, indicating that the direct effect of parental punishment on social retreat is not significant, The effect value is 0.04, the path of the mediation effect is parental punishment → emotional regulation → social withdrawal, The 95% confidence interval of the path coefficient is 0.01–0.06, which does not include 0, indicating that the indirect effect of parental punishment on the social withdrawal through emotional regulation is significant (Table 7).

**Table 7:** Test of the mediating effect of emotional regulation between parental punishment and social withdrawal.

Path	Value	SE	Weight (%)	95% CI	
				Max	Min
Parental punishment → Social withdrawal	0.04	0.03	57.14	−0.02	0.09
Parental punishment → Emotional regulation → Social withdrawal	0.03	0.02	42.86	0.01	0.06
Overall	0.07	0.03	100.00	0.01	0.13

#### 4.4.2 The mediating role of emotion regulation between parental preference and social withdrawal

We use Model 4 in the SPSS macro-program and examine the mediating role of emotion regulation between parental preference and social withdrawal, and the results are shown in Table 8.

**Table 8:** Test results of the mediation model of emotional regulation between parental preference and social withdrawal.

Predictors	Outcome variables	Fit		Sig.	
		*R* ^2^	*F*	*t*	β
Preference	Social withdrawal	0.01	4.07	2.02*	0.07
Preference	Emotional regulation	0.05	13.90	−3.73***	−0.15
Emotional regulation	Social withdrawal	0.20	33.89	−7.92***	−0.39
Preference				0.44	0.01

*p < 0.05, **p < 0.01, ***p < 0.001.

The results showed that parents preferred positive prediction of social retreat ( β=0.07 , p<0.05 ) and negative prediction of emotional regulation ( β=0.15 , p<0.001 ); Parents prefer to enter the equation at the same time as emotional modulation ability ( β=−0.39 , p<0.001 ) to predict social retreat negatively.

The bias-corrected percentile Bootstrap test shows that, as shown in Table 7, the 95% confidence interval for parental preference is 0.05–0.08, including 0, indicating that parental preference has no significant direct effect on social retreat. The effect value is 0.01, and the path of the mediation effect is parental preference → mood regulation → social retreat; the 95% confidence interval of the path coefficient is 0.02–0.09 and does not include 0, indicating that the indirect effect of parents’ preference to affect social retreat through emotional regulation is significant (Table 9).

**Table 9:** Test of the mediating effect of emotion regulation between parental preference and social withdrawal.

Path	Value	SE	Weight (%)	95% CI	
				Max	Min
Preference → Social withdrawal	0.01	0.03	14.29	−0.05	0.08
Preference → Emotional regulation → Social withdrawal	0.06	0.02	85.71	0.02	0.09
Overall	0.07	0.04	100.00	0.01	0.14

#### 4.4.3 The mediating role of emotional regulation between parental rejection and social withdrawal

We use Model 4 in the SPSS macro-program and examine the mediating role of emotion regulation between parental rejection and social withdrawal, as shown in Table 10.

**Table 10:** Mediation model test results of emotion regulation between parental rejection and social withdrawal.

Predictors	Outcome variables	Fit		Sig.	
		*R* ^2^	*F*	*t*	β
Rejection	Social withdrawal	0.03	8.02	2.83**	0.14
Rejection	Emotional regulation	0.05	14.53	−3.81***	−0.21
Emotional regulation	Social withdrawal	0.20	34.83	−7.74***	−0.38
Parental punishment				1.31	0.06

*p < 0.05, **p < 0.01, ***p < 0.001.

The results show that parents refuse to predict positive social retreat (β = 0.14, *p* < 0.05) and negative predictive emotional regulation (β = 0.21, *p* < 0.001); Parents refuse to enter the equation at the same time as their ability to regulate emotions, and their ability to regulate emotions (β = −0.38, *p* < 0.001) negatively predicts social retreat.

The bias-corrected percentile Bootstrap test shows that, as shown in Table 9, the confidence interval of 95% of the rejection path coefficient is 0.03–0.15, including 0, indicating that the direct effect of the rejection on social retreat is not significant. The effect value is 0.06, and the path of the mediation effect is parental rejection → emotional regulation → social retreat; the 95% confidence interval of the path coefficient is 0.03–0.12, excluding 0, indicating that the indirect effect of parents’ refusal to influence social retreat through emotional regulation is significant.

## 5 Discussions and conclusions

### 5.1 Variances in parental rearing, emotional regulation, and social withdrawal among upper primary school children

The present study discerned notable disparities across the three dimensions of parental home-rearing practices—namely, rejection, overprotection, and emotional warmth—when comparing different grades. Notably, fifth-grade students exhibited significantly higher levels in these dimensions compared to their fourth and sixth-grade counterparts. This finding aligns with the extant literature, which posits that parental tendencies toward punishment, rejection, and intrusive behavior generally diminish with the child’s advancing age. However, an observed increase in these behaviors during the fifth grade may be attributed to the transitional challenges children face as they progress from the fourth to the fifth grade, potentially leading to increased stress and a concomitant rise in behavioral and learning difficulties. This phenomenon may prompt parents to heighten their engagement in their children’s developmental and educational processes.

Furthermore, the study revealed that fifth-grade students demonstrated a markedly lower capacity for emotional regulation compared to their peers in the fourth and sixth grades. The heightened level of social withdrawal in the fifth grade can be attributed to the critical developmental period that spans these grades, where children’s self-awareness escalates, particularly around the age of 10. During this juncture, children exhibit an intensified need for respect and understanding from their parents, while simultaneously desiring autonomy and liberation from parental oversight. The escalating academic pressures and the requisite adaptation phase for both children and parents contribute to the pronounced social withdrawal and diminished emotional regulation observed in fifth-grade students. Conversely, as students advance to the sixth grade, maturation processes ensue, and their competencies in problem-solving and emotional modulation are observed to improve. Consequently, sixth-grade students exhibit superior emotional regulation abilities and reduced levels of social withdrawal compared to those in the fifth grade.

Our results suggest that parenting styles likely influence social withdrawal through their impact on children’s sense of autonomy, competence, and relatedness, as proposed by SDT (Ryan and Deci, 2017). Future research could explore this mediating effect in more detail. For instance, studies could examine how specific parenting behaviors (e.g., offering choices vs. micromanaging) influence children’s perceptions of autonomy and how these perceptions, in turn, relate to social engagement.

### 5.2 Investigation of home-schooling, emotional regulation and social withdrawal in upper primary school children

This study’s findings elucidate the intricate relationships between parental rearing practices, children’s emotional regulation, and the incidence of social withdrawal.

#### 5.2.1 Positive correlation between parental warmth and emotional modulation

Initially, it was observed that a significant positive correlation exists between the emotional warmth demonstrated by parents and their offspring’s capacity for emotional modulation. Conversely, parental practices such as punishment, rejection, and preferential treatment were found to be negatively associated with children’s ability to modulate emotions. This aligns with a body of literature suggesting that home-rearing environments can significantly influence the emotional regulatory strategies employed by children. Parental warmth often fosters an affirming environment that encourages the development of self-esteem and self-confidence in children, thereby facilitating the maintenance of positive affect and the circumvention of negative emotional states. Timely parental guidance is also highlighted as a critical factor in enhancing children’s emotional regulatory competencies. In contrast, the imposition of psychological stress through punitive, rejecting, or preferential behaviors can precipitate anxiety and other emotional disturbances that may exceed the child’s self-regulatory capacity.

#### 5.2.2 Parental behaviors and social withdrawal

Subsequently, the study revealed a positive correlation between parental punishment, rejection, and preferential treatment, and the social withdrawal of children. This finding is congruent with prior research, underscoring the pivotal role parents play in the social dynamics of their children. When parents engage in punitive and rejecting behaviors, the emotional needs of children may be neglected, leading to aversive experiences and the deployment of psychological defenses that culminate in social withdrawal. Additionally, preferential treatment and inadequate parental care can impair children’s ability to discern and navigate the veracity of external stimuli.

#### 5.2.3 Emotional regulation and social withdrawal

Lastly, the research established a significant negative correlation between children’s emotional regulation abilities and social withdrawal behavior. The robustness of a child’s emotional regulatory capacity was found to inversely predict the extent of their social retreat. This is in concordance with existing research, which posits that children adept at mood regulation are better equipped to manage their negative emotions, thereby circumventing anxiety, depression, and agitation, and reducing the propensity for social withdrawal. Emotional regulation is thus posited as a critical component for children’s social development, with dysregulation potentially leading to a myriad of behavioral issues that can impede cognitive and social functioning.

### 5.3 The mediating role of emotional regulation

The study’s findings articulate that the mechanisms of emotional regulation serve as an intermediary in the relationship between family rearing practices and social withdrawal behaviors. Specifically, it was observed that elements of parental rearing, such as punishment, rejection, and preferential treatment, indirectly shape a child’s social withdrawal through their capacity for emotional regulation. This mediation effect suggests that the deleterious effects of certain parental behaviors on children’s social engagement are transmitted, at least in part, through their impact on children’s emotional regulatory abilities.

This conclusion aligns with a body of literature indicating (Morris et al., 2017; Wu et al., 2021). that the intensity of parental rearing practices can exert both direct and indirect influences on the social and emotional development of children, with emotional regulation being a pivotal mediating factor. It implies a reciprocal relationship where increased instances of parental punishment, rejection, and favoritism are associated with diminished emotional regulation skills in children, thereby escalating the propensity for social withdrawal.

The accumulation of negative emotions as a consequence of punitive and dismissive parenting can engender emotional distress in children, undermining their capacity for effective emotional regulation. This, in turn, predisposes children to develop distorted perceptions and adverse experiences regarding self and environment, culminating in an impediment to the acquisition of normative social competencies and an inclination toward social retreat.

Thus, the study underscores the criticality of children’s emotional regulation as a mediational fulcrum linking parental practices and the phenomenon of social withdrawal. It highlights the necessity for parents to foster an emotionally supportive environment that bolsters children’s emotional regulatory skills, which are integral not only to their immediate socio-emotional well-being but also to their long-term social integration and personal development.

### 5.4 Suggestions

After the above analysis, we find that parental rearing patterns directly affect children’s emotions. If parents give children tenderness, support, positivity, and other positive emotions, the child will also grow healthier. At the same time, parents also need to be aware that children should not be overly restrained. Too much interference in the lives of children will also cause children to develop various problems. Therefore, parents should take appropriate measures in the latter years of education. Based on this, this paper puts forward the following suggestions for parental education:

1. Strengthen emotional control: In this stage of child development, some children, due to their low level of cognition, better control their emotions, and there are moody and erratic situations. Therefore, parents should exercise specific emotional control and strengthen the restraint, supervision, and discipline of children. However, parents should be gentle, patient, and teaching-based during this stage, not over-punishing and controlling. In the adolescent stage, the cognition level of the adolescent has improved significantly. And the physiological level tends to mature. Adolescents pursue autonomy and freedom at this stage, but their psychological development remains immature. Therefore, parents should be instructed and restrained accordingly. But control is more broad than childhood. It is reasonable to control ethical, social, and security issues. As well as personal preferences, teenagers will have their own unique views. Parents should not unduly interfere. In order to ensure the safety of adolescents, adolescents can make their own choices.
2. Psychological control: parents should not exert excessive psychological control on children or adolescents, which may cause the development of the child’s independence and autonomy to be adversely affected, resulting in the child being weak, lonely, dependent on the parents, lack of independence, unable to accomplish the corresponding affairs themselves. Psychological control of the child should, therefore, be avoided as much as possible.
3. Parents should express their concern and thoughtfulness. It is important to note that at any stage, parental support is vital for children, which can improve their self-confidence, enable children to have positive emotions such as self-esteem and self-love and strengthen their ability to interact and regulate emotions.

### 5.5 Limitations and future research

Although we have an influential analysis of the mediating effect of emotional regulation, we still have limitations in this study. We use students from several schools in a province in eastern China as a sample, and it is recommended that future studies focus on students from schools in different categories and regions and even cross-country comparisons between other countries to obtain more conclusions. Second, the study was conducted in a cross-sectional design based on a self-reported questionnaire and could not infer causal relationships between variables. Future studies will focus on the usage of a mediating model with a longitudinal design to understand the associations between variables better.

In future studies, the genetic quality and cognitive development of children can also affect the level and condition of emotional regulation. Despite our emphasis on social and cultural factors, the children’s own genetic material must be considered as the material basis for emotional regulation.

Furthermore, in light of the current findings and the recent work by Ahmadi et al. (2023), there is a clear avenue for future research to develop and rigorously test the effectiveness of intervention programs aimed at parents. Such programs could focus on enhancing parents’ need-supportive behaviors, with the ultimate goal of improving their children’s emotional regulation and social development.

Specifically, future research could: Design intervention programs based on the classification system of need-supportive behaviors proposed by Ahmadi et al. (2023), tailoring the intervention to the specific needs and contexts of upper primary school children; Implement randomized controlled trials to evaluate the effectiveness of these interventions in improving parental behaviors and, consequently, children’s emotional regulation and reduction in social withdrawal; Explore the long-term effects of such interventions on children’s mental health, academic performance, and overall well-being.

The development of these interventions should consider the cultural, social, and individual differences among families to ensure broad applicability and effectiveness. By focusing on practical, evidence-based strategies for parents, we can contribute to the burgeoning body of research that seeks to optimize children’s emotional and social development.

## Data availability statement

The raw data supporting the conclusions of this article will be made available by the authors, without undue reservation.

## Ethics statement

This study was conducted in accordance with the guidelines of the Declaration of Helsinki and was approved by the Ethics Committee of Shenyang Normal University. The studies were conducted in accordance with the local legislation and institutional requirements. Written informed consent for participation in this study was provided by the participants’ legal guardians/next of kin.

## Author contributions

TY: Conceptualization, Data curation, Formal analysis, Funding acquisition, Investigation, Methodology, Project administration, Resources, Software, Supervision, Validation, Visualization, Writing – original draft, Writing – review & editing. ZM: Conceptualization, Investigation, Software, Writing – original draft, Writing – review & editing. YZ: Conceptualization, Writing – original draft, Writing – review & editing.

## Footnotes

^1^Swedish acronym for Egna Minnen Beträffande Uppfostran.
